# Emphasis on prevention: how to approach residual cardiovascular risk

**DOI:** 10.1007/s12471-021-01643-x

**Published:** 2021-11-02

**Authors:** J. H. Cornel, R. J. G. Peters

**Affiliations:** 1 Department of Cardiology, Radboud University Medical Centre, Nijmegen, The Netherlands; 2 Department of Cardiology, Northwest Clinics, Alkmaar, The Netherlands; 3 Department of Cardiology, Amsterdam University Medical Centres, location AMC, Amsterdam, The Netherlands

Despite huge efforts to prevent recurrent atherosclerotic cardiovascular disease (ASCVD), in many patients the risk of complications remains very high. A number of factors contribute to this ‘residual risk’. These include insufficient lifestyle improvements and insufficient compliance with recommended treatments, both by patients and physicians. In addition, even when guideline-based preventive measures are completely implemented, a significant risk of recurrent events remains. Apparently, our current treatment options are unable to completely suppress the underlying disease. This special issue of the *Netherlands Heart Journal *is dedicated to prevention of ASCVD, including the challenges of ‘residual risk’.

Lifestyle management is the cornerstone of both primary and secondary prevention of atherosclerotic cardiovascular disease and the importance of lifestyle management is emphasised by all major guidelines. However, implementation of lifestyle management is difficult and often not successful. Van Trier et al. [1] stress the responsibility of healthcare professionals to address unhealthy lifestyle habits and to support a change towards healthy habits. Achieving meaningful and persisting lifestyle changes is challenging and possibly better outcomes are achieved when behavioural change is incorporated into daily life and in line with personal patient values.

Residual lipid-driven, inflammatory and thrombotic risks in ASCVD are another opportunity to prevent major adverse cardiovascular events, requiring individual profiling and personalised medicine. Lipid-driven cardiovascular disease risk is caused by atherogenic apoB particles containing low-density lipoprotein cholesterol, triglycerides and lipoprotein(a) and represents an important and modifiable part of the total cardiovascular risk. Nurmohamed and Stroes [2] describe the latest developments with many novel lipid-lowering therapies in this exciting research field and they try to convince the readers that we will be able to achieve guideline-advised, lipid-driven targets more successfully in the near future. Importantly, a personalised treatment dependent on absolute CVD risk and respective lipid profile should be configured. Fiolet et al. [3] point out how the inflammatory biology of atherosclerosis has translated into clinical therapeutic options. The low-dose colchicine studies are the most clinically relevant recent development, showing a consistent and clinically relevant relative risk reduction for major cardiovascular events in acute and chronic coronary syndromes without paying a price in terms of safety risk. These findings could be the beginning of the use of anti-inflammatory therapy in cardiovascular disease. The struggle with how to define high residual thrombotic risk, how to balance the increased bleeding risk and to choose the best therapeutic option for a particular patient is reviewed extensively by Chan Pin Yin and ten Berg [4] and a personal preference is described by the authors.

The PANORAMA working group describe their attempt to provide guidance for clinicians focusing on absolute risk assessment and individual risk profiling [5]. Shared decision-making will hopefully enhance patient compliance. Other aspects, such as competing risk and quality of life, should also be considered. Novel treatment options, with the exception of the generic drug colchicine, are often expensive, necessitating careful selection of patients to ensure cost efficacy of these interventions. Interesting initiatives are emerging, among which are the lifetime risk and benefit calculators. The introduction of artificial intelligence could help clinicians and patients to make the best decision in tailored therapy in cardiovascular prevention.

Finally, the government has an important task to educate the population and to stimulate a healthy lifestyle. This special issue finishes with a strong appeal to politicians to take the opportunities to decrease avoidable loss of healthy life years [6]. The COVID 19 pandemic has paved the way to the long-awaited next level of government interference.
